# Poor Adherence to the Mediterranean Diet and Sleep Disturbances Are Associated with Migraine Chronification and Disability among an Adult Population in the Lazio Region, Italy

**DOI:** 10.3390/nu16132169

**Published:** 2024-07-08

**Authors:** Roberta Bovenzi, Annalisa Noce, Matteo Conti, Manuela Di Lauro, Barbara Chiaramonte, David Della Morte, Alessandro Stefani, Antonino De Lorenzo, Nicola Biagio Mercuri, Maria Albanese

**Affiliations:** 1 Department of Systems Medicine, University of Rome Tor Vergata, 00133 Rome, Italy; roberta.bovenzi@gmail.com (R.B.); annalisa.noce@uniroma2.it (A.N.); matteoconti92@gmail.com (M.C.); stefani@uniroma2.it (A.S.); mercurin@med.uniroma2.it (N.B.M.); 2 UOSD Nephrology and Dialysis, Tor Vergata University Hospital, 00133 Rome, Italy; dilauromanuela@gmail.com; 3 Istituto Nazionale per l’Assicurazione Contro Gli Infortuni sul Lavoro (INAIL), Actuarial-Statistic Consultancy Office, Via Stefano Gradi, 55, 00143 Rome, Italy; b.chiaramonte@hotmail.com; 4 Section of Clinical Nutrition and Nutrigenomic, Department of Biomedicine and Prevention, University of Rome Tor Vergata, 00133 Rome, Italy; dmorte@med.miami.edu (D.D.M.); delorenzo@uniroma2.it (A.D.L.); 5 Department of Neurology, Evelyn F. McKnight Brain Institute, University of Miami Miller School of Medicine, Miami, FL 33136, USA; 6 Parkinson’s Disease Unit, Tor Vergata University Hospital, 00133 Rome, Italy; 7 Faculty of Medicine and Surgery, University of “Nostra Signora del Buonconsiglio” UnizKm, 1000 Tirana, Albania; 8 Regional Referral Headache Center, Neurology Unit, Tor Vergata University Hospital, 00133 Rome, Italy

**Keywords:** migraine, lifestyle, diets, Mediterranean diet, sleep, physical exercise

## Abstract

Lifestyle factors, such as diet and sleep quality, are receiving increasing interest as accessible therapeutic approaches to migraine. The Mediterranean diet (MD) has shown clear benefits in cardiovascular and metabolic diseases, as well as in sleep patterns. Here, our objective was to identify the impact of adherence to the MD and other lifestyle factors on the clinical burden of migraine. For this purpose, we enrolled 170 migraine patients and 100 controls, assessing the clinical disability of headache using standardized clinical scales (HIT-6 and MIDAS) in the migraineur cohort and lifestyle patterns in both groups through the PREDIMED score for MD adherence, the IPAQ scale for physical activity, and BMI. Subjects were also screened for sleep–wake disturbances based on the Pittsburgh Sleep Quality Index (PSQI). We found that migraine patients had lower adherence to the MD compared to the controls and that the HIT-6 scale had a significant negative relationship with MD adherence in patients with high-frequency episodic and chronic migraine. Additionally, in the same migraine patients, the presence of sleep–wake disturbances was correlated with greater migraine disability as assessed by the MIDAS score. In conclusion, this study found that among different lifestyle factors, poor adherence to the MD and the presence of sleep–wake disturbances were closely associated with migraine disability and chronification.

## 1. Introduction

Migraine is defined as a complex neurovascular disorder characterized by recurrent attacks of disabling pain, accompanied by nausea, vomiting, phonophobia, photophobia, and sensitivity to movement, lasting for 4–72 h [1]. It is a highly prevalent public health concern which afflicts about one billion young adults worldwide, creating substantial personal and socioeconomic burdens [2,3].

Episodic migraines occur sporadically, typically for less than 15 days a month, while chronic migraines are characterized by headaches occurring on 15 or more days per month for at least three months, with the headaches on at least 8 of those days being migraines. This distinction is crucial because chronic headaches often require additional and more aggressive preventive approaches to reduce their frequency and impact on daily life. Furthermore, episodic migraine sufferers convert into chronic migraine sufferers with an approximate annual rate of 3%, and this is expected to triple by 2050 unless preventive strategies are developed [4]. Due to its intermittent nature and clinical variability in each patient, identifying the modifiable factors that may predict this transformation is a key factor in migraine management.

In this regard, increasing evidence suggests that proper nutrition and a healthy lifestyle could have the potential to affect the clinical course of migraine due to their pivotal role in systemic inflammation, cerebral vessel vasodilation, and cerebral glucose metabolism [5]. Various foods and dietary components, such as alcohol, caffeine, and chocolate, can trigger migraine attacks, and migraine symptoms can be mitigated by healthy eating behaviors [6].

Over the past few years, adherence to certain dietary patterns, such as the Mediterranean diet (MD), has shown clear benefits in terms of total mortality and the primary and secondary prevention of non-communicable chronic diseases, including obesity, arterial hypertension, diabetes mellitus, metabolic syndrome, and neurological disorders, due to its proven anti-inflammatory and cardioprotective effects [7,8,9,10]. The MD has also shown promising associations with lower migraine severity and better sleep characteristics, although the results of these studies have not been conclusive, and evidence for a causal relationship is limited due to the small number of diet intervention/epidemiological studies conducted [11,12]. Furthermore, the majority of them have focused on the effects of dietary triggers on migraine and the sleep–wake cycle, while the way migraine is associated with adherence to diet and sleep dysfunction has received less attention.

In this sense, it is important to identify whether the subtypes of migraine, namely episodic and chronic migraines, can be influenced by these factors. Therefore, the objective of this study was to explore the direct effect of adherence to MD patterns and other lifestyle habits, specifically physical activity, weight status, and sleep, on headache features and disability among a population of episodic and chronic migraine patients and in a cohort of controls.

## 2. Materials and Methods

### 2.1. Subjects

This single-center cross-sectional study involved 170 subjects diagnosed with migraine and followed up at the Regional Headache Referral Center of the University of Tor Vergata, Rome. The inclusion criteria included (1) patients of both sexes, of legal age (>18 years), and (2) with a diagnosis of migraine made according to the criteria of the International Headache Society (IHS ICHD-3, 2018) [1]. The exclusion criteria were (1) a history of other neurological or psychiatric conditions; (2) altered consciousness, organ failure, or severe infectious diseases that could have precluded the clinical pathway; (3) the presence of internal disorders (history of renal, thyroid, or liver disease, cancer, diabetes, cardiovascular disorders); (4) the use of nutritional and herbal supplements (e.g., magnesium, riboflavin, etc.); (5) unavailability to fill in self-administered clinical scales. As a control group, we enrolled 100 subjects of the same age and sex without any history of neurological, psychiatric, or internal conditions or cancer. These subjects were specifically recruited for this purpose. The study was carried out according to the guidelines of the Declaration of Helsinki and was approved by the Ethics Committee of the University of Rome, Tor Vergata (protocol number 85/20). Written informed consent was obtained from all the subjects involved in the study.

### 2.2. Evaluations of Demographic and Clinical Characteristics in the Migraine Patient Cohort

All patients underwent detailed medical history collection related to their main demographic and clinical characteristics (age, sex, comorbidities, concomitant therapies) and the clinical features of their migraine episodes: time since migraine diagnosis, monthly migraine days (MMDs), mean attack duration, number of symptomatic drugs taken, location and type of pain (pulsating, sharp, or other), the presence of accompanying unilateral autonomic symptoms (UASs), previous preventive medications, number of preventive treatments failed (more than 3 previous preventive treatments failed: yes or no), and response to triptans (no, scarce, good, excellent). Specifically, the response to triptans, 5HT-1B/1D receptor agonists developed to treat acute migraine, was included as these symptomatic agents appear to induce migraine progression in individuals with high-frequency migraines (8–14 days per month) and to be protective in those with <8 days of headache at baseline [13].

Furthermore, the following three validated headache impact tools were used to assess the disability and severity of migraine:

- The Migraine Disability Assessment Scale (MIDAS) questionnaire, the most used disability instrument in migraine research based on five questions that focus on lost time in three domains: schoolwork or work for pay; household work or chores; and family, social, and leisure activities [14]. All questions ask about days of missed activity or days where productivity was at least halved. If productivity is reduced to 50% or below, the day is considered missed. The MIDAS score is derived as the sum of days missed due to a headache over a 3-month period in the three domains [14];
- The Headache Impact Test-6 (HIT-6), a self-administered six-item questionnaire that measures the impact of headache on “usual daily activities”, including work, school, or social activities, assessing the severity of pain, fatigue, the desire to lie down, frustration, and difficulty concentrating. The HIT-6 questionnaire has good discriminative validity, internal consistency (Cronbach’s α of 0.79), and test–retest reliability (average Cronbach’s α of 0.78) [15];
- The visual analogue scale (VAS) for the evaluation of pain intensity. It is presented graphically with a 10 cm line and endpoint adjective descriptors (“the worst imaginable pain” on one end and “no pain” on the other). The patient is asked to place a mark along the line to indicate their current level of pain. A difference of 13 mm between consecutive pain ratings is the minimum change in a clinically significant pain rating [16].

### 2.3. Evaluations of Lifestyle Habits and Diet Patterns in Migraine Patients and Controls

A validated 14-item questionnaire, the “PREvención con DIeta MEDiterránea” (PREDIMED), was administered by the research staff to all migraineurs and controls [17]. PREDIMED is a well-established tool commonly used in Mediterranean basin studies to assess long-term adherence to the MD. The scale consists of 12 questions focused on specific food quantities and the daily or weekly frequency of consumption (olive oil, vegetables, fruit, red or processed meats, butter, soda drinks, legumes, fish, commercial sweets, nuts, wine, sofrito sauce) and 2 general questions on olive oil and meat intake habits. Each item offers binary answer choices with corresponding scores: 1 for “yes” and 0 for “no”. Based on their total PREDIMED scores, participants were categorized into three distinct MD adherence groups: poor adherence (score ≤ 5), moderate adherence (score between 6 and 9), and good adherence (score ≥ 10). All subjects were asked to report their level of physical activity over the past seven days using the validated International Physical Activity Questionnaire (IPAQ) [18]. According to this questionnaire, subjects were rated as inactive (IPAQ score < 700), sufficiently active (IPAQ score between 700 and 2519), and very active (IPAQ score ≥ 2520). Body mass index (BMI), determined by dividing weight (kg) by height squared (m^2^), was calculated for all subjects to assess their weight status.

Lastly, detailed medical history was collected from each subject to identify coexisting insomnia or other circadian rhythm sleep–wake disorders and coupled with the validated Pittsburgh Sleep Quality Index (PSQI), a questionnaire specifically designed to measure sleep quality [19]. The PSQI includes 19 items according to seven components, namely subjective sleep quality, sleep latency, sleep time, habitual sleep efficiency, sleep disorders, sleep medication, and daytime dysfunction. A validated cut-off score of 5 indicates poor sleep quality [19]. Based on this cut-off score, migraine sufferers were classified as having comorbid sleep–wake disturbances (PSQI ≥ 5) or not (PSQI < 5) [19].

### 2.4. EM^LF^ and EM^HF^+CM Groups

We then divided the cohort of migraine patients into two subgroups: patients with low-frequency episodic migraine (EM^LF^, <8 days of migraine/month) and patients with high-frequency episodic migraine (8–14 days of migraine/month) and with chronic migraine (≥15 days of migraine/month) (EM^HF^+CM). Patients with EM^HF^ and CM were considered together, according to a continuum of disease severity in which patients have frequent and spontaneous fluctuations between these two conditions [20]. Additional subgroup analyses were not performed due to the limited number of patients, potentially reducing the statistical power of the results.

### 2.5. Statistical Analysis

A two-sample T-test was used to analyze the differences in the quantitative demographic and clinical variables between groups. The Chi-square test was used to compare the qualitative (categorical) clinical characteristics between groups. The relationships between migraine characteristics (MIDAS, HIT-6, VAS, MMDs, and mean attack duration) and lifestyle factors (IPAQ, PREDIMED, and sleep–wake disturbances) were analyzed using generalized linear models (GLMs), using age, sex, and BMI as covariates. In particular, we used logistic regression to analyze the relationship between each clinical migraine characteristic and the presence of sleep–wake disturbances based on the PSQI scale (Y/N binomial variable) and ANCOVA to compare each migraine characteristic across different categories of IPAQ and PREDIMED scores. Post hoc analysis based on Tukey’s method was used for ANCOVA. The GLMs were analyzed separately within each subgroup of migraine patients (EM^LF^ and EM^HF^+CM). Statistical significance was established at *p* < 0.05. All the statistical analyses were performed using SPSS 25.0 statistical software.

## 3. Results

### 3.1. Cohort Characteristics

This study involved 170 migraine patients (mean age **=** 44.4 ± 13.3, 18.23% males and 81.77% females) and 100 controls. Table 1 shows the main demographic and clinical characteristics of the study population.

### 3.2. Migraine Patient Characteristics

The patients experienced an average of 8.2 ± 7.6 days of migraine per month, took 5.3 ± 7.3 painkillers per month, and had a mean level of disability corresponding to a MIDAS score of 18.8 ± 22.5 and a HIT-6 score of 57.6 ± 9.3. In terms of pain location, most of the patients reported fixed unilateral (n = 50, 29.4%) or bilateral pain (n = 82, 48.2%). Regarding the type of pain, n = 79 patients (46.5%) reported suffering from pulsating pain, n = 3 (1.8%) from sharp pain, and n = 88 (51.7%) from another type of pain. Only n = 19 patients (11.2%) reported the presence of accompanying UASs. The patients were mostly treated with one (n = 87, 51.2%) pharmacological preventive treatment, and only n = 12 (7.1%) had failed more than three different preventive classes of drugs. A total of n = 41 patients (24.1%) did not receive any preventive medication. Additionally, the patients had a mean duration of attack of 34.9 ± 20.7 h, with a medium intensity as assessed by a VAS score of 8.1 ± 1.8. The patients reported having a mostly good (n = 75, 44.1%) or excellent (n = 63, 37.1%) response to triptans. Regarding sleep–wake disturbances, n = 110 patients reported suffering from some form of circadian sleep–wake rhythm disturbance (64.7%) (PSQI > 5), while n = 60 did not (35.3%) (PSQI ≤ 5) (Table 1).

### 3.3. Lifestyle Habits and Dietary Patterns in Migraine Patients and Controls

Both the patients and the controls in our cohort had a mean normal weight (BMI = 23.6 ± 3.5 vs. 24.1 ± 4.2).

The PREDIMED scores differed significantly between the migraine patients and controls (*X*^2^: 14.10, *p* < 0.001). In particular, migraine patients appeared to have poorer adherence to the MD compared to the controls (Table 1). Furthermore, in our study population, migraine patients were more physically active than the controls, as assessed by the IPAQ score (*X*^2^: 9.79, *p* = 0.007) (Table 1). No further differences emerged between the migraineurs and controls regarding sleep dysfunction (Table 1).

### 3.4. EM^LF^ and EM^HF^+CM Subgroup Features

Table 2 contains the main demographic and clinical characteristics of the migraineurs as classified according to the frequency of pain: EM^LF^ patients (n = 98, 57.6%) and EM^HF^+CM patients (n = 72, 42.4%). The two subgroups were homogeneous in terms of age, disease duration, and BMI.

As expected, the patients with EM^HF^+CM used a higher number of symptomatic treatments per month (t = 6.7, *p* < 0.001) and reported higher VAS (t = 3.1, *p* = 0.002), MIDAS (t = 3.2, *p* = 0.002), and HIT-6 scores (t = 7.6, *p* < 0.001) compared to patients with EM^LF^ (Table 2). Note that among these headache impact tools, HIT-6 score was the variable that differed between the groups with the highest statistical significance.

The number of preventive treatments (*X*^2^ = 11.7, *p* = 0.019) and the response to triptans (*X*^2^ = 14.1, *p* = 0.003) differed significantly between the two subgroups (Table 2). On the contrary, IPAQ and PREDIMED scores, pain localization and type, the presence of UASs, and sleep–wake disturbances did not differ between the two subgroups.

### 3.5. Relationships between Migraine Characteristics and Lifestyle Factors in the EM^LF^ and EM^HF^+CM Subgroups

At this point, we created GLMs using each quantitative clinical feature of migraine (MIDAS, HIT-6, and VAS scores, MMDs, and mean attack duration) as dependent variables and IPAQ and PREDIMED scores and sleep–wake disturbances as independent variables in each group, using age, sex, and BMI as covariates.

In the EM^LF^ group, no significant differences were found in any clinical feature of migraine (MIDAS, HIT-6, and VAS scores, MMDs, or mean attack duration) among the three PREDIMED and IPAQ scores.

**Figure 1:**
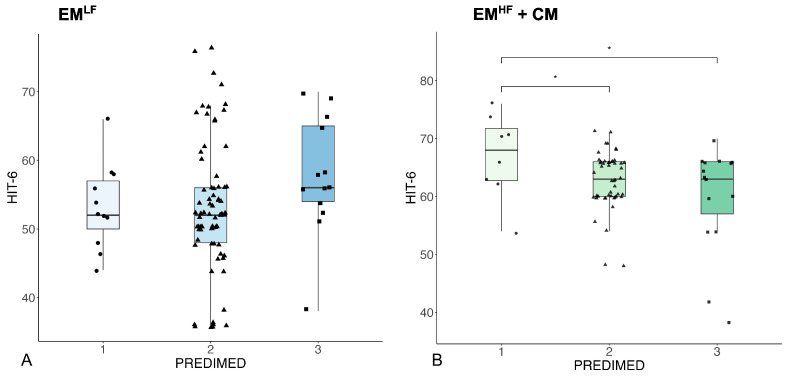
This figure shows the lack of statistically significant differences between the HIT-6 variable among the three PREDIMED scores in the EM^LF^ group (picture **A**) and the significant difference found in the HIT-6 variable between PREDIMED 1 and 2 (* *p* = 0.037) and between PREDIMED 1 and 3 (* *p* = 0.016) in the EM^HF^+CM group (picture **B**).

In the EM^HF^+CM group, we found a significant difference between the HIT-6 scores among the three PREDIMED scores (F = 5.79, *p* = 0.017) (Figure 1, graph B). In particular, the post hoc analyses showed a significantly higher HIT-6 score in PREDIMED 1 (poor adherence) compared to 2 (moderate adherence) (*p* = 0.037) and 3 (good adherence) (*p* = 0.016) (Figure 1, plot B) but not between PREDIMED 2 and 3.

**Figure 2:**
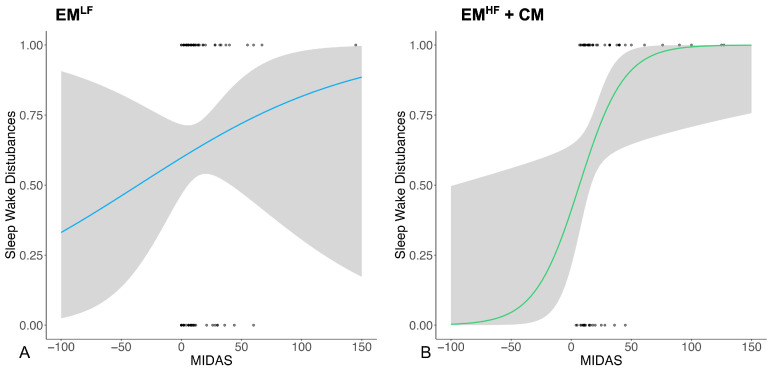
This figure shows the lack of a significant association found in the EM^LF^ group (**A**) and the significant positive logistic relationship between the presence of sleep–wake disturbances (1 = yes, 0 = no) and the MIDAS scores in the EM^HF^+CM group (**B**).

Using logistic regression, we then found that the presence of sleep disturbances was associated with higher MIDAS scores only in the EM^HF^+CM group (r^2^ = 0.79, *p* = 0.017) (Figure 2, graph B), while no significant associations were found in the EM^LF^ group (Figure 2, graph A). No further associations were found between the presence of sleep–wake disturbances and other migraine characteristics in the two groups.

## 4. Discussion

This study aimed to investigate the impact of lifestyle patterns on migraine characteristics in a wide cohort of patients with migraine.

Among diet patterns, physical activity, BMI, and sleep quality, we found that both adherence to the MD and sleep disturbances had a significant impact on migraine frequency and disability.

Lifestyle patterns are receiving increasing interest in headache management due to their potential role in mitigating the severity of migraine with little cost and risks for patients [21].

Diet is an important lifestyle factor that can be modulated to potentially impact migraine. However, despite compelling evidence supporting the beneficial role of specific diet patterns such as the ketogenic diet [22], no single “one-size-fits-all” diet has yet been identified as the best strategy for migraine sufferers [21]. However, it is well known that migraine can be aggravated in some patients by fasting, alcohol intake, excessive caffeine consumption, insufficient B vitamins, excessive sugar intake, and other unhealthy dietary habits.

In this study, we focused on the MD, a traditional eating habit in countries bordering the Mediterranean Sea [23]. The MD is much more than a simple diet. In fact, it is a real lifestyle that encourages correct eating habits and conviviality.

The MD is characterized by the daily consumption of plant-based foods, such as fruit, vegetables, nuts, and legumes; moderate consumption of foods of animal origin, such as lean meat, dairy products, and fish; and the use of extra virgin olive oil as the main source of fat [23,24,25,26,27]. The MD is rich in antioxidants, trace elements, minerals, and vitamins that modulate inflammatory and oxidant pathways [28,29].

Here, we rated adherence to the MD based on the validated 14-item PREDIMED questionnaire, first used by the Spanish trial “PREvención con Dieta MEDiterránea” trial [19], which distinguishes three grades of adherence (poor, moderate, and good), and related it to migraine characteristics.

First, we found that approximately 16% of the subjects had good adherence to the MD. This percentage appeared to be slightly higher than those described in the literature, with around 6% to 14% of Italian subjects reported as having strong adherence to the MD [30,31], still lower than the 29% rate we found in the control group involved in this study, further suggesting a role of the MD in migraine prevention.

In fact, as the main finding of this study, we found a significant relationship between higher adherence to the MD and lower migraine disability, as assessed by the HIT-6 score, among patients with high-frequency episodic and chronic migraine.

High-frequency episodic migraine is a condition at higher risk of converting into CM [32], the most disabling migraine condition, characterized by persistent headaches occurring on at least 15 days each month, with migraine-like features on at least 8, for more than three months [1]. CM imposes a considerable burden on individual and socioeconomic outcomes. People with EM can remit, not change, or progress to CM over time [33]. The transformation from EM to CM occurs in approximately 3% of those with EM annually [33]. However, not all high-frequency EM evolves into CM; therefore, it is important to identify those at high risk of chronification. Among the risk factors, the identification of modifiable factors is important for implementing behavioral and pharmacologic interventions that could delay the risk of conversion into CM [33].

On the other hand, CM is a condition characterized by the breakdown of peripheral and central nociceptive networks [34], which drives an abnormal response to sensory inputs below the threshold, thus maintaining a vicious cycle that can eventually become independent from external triggers [35,36]. Therefore, high-frequency EM represents the optimal window to intervene with preventive strategies, including lifestyle modifications and diet patterns.

Here, we found that adherence to the MD was associated with a reduced disability burden in those patients with CM and high-frequency EM, those at higher risk of converting into CM, thus suggesting that adherence to the MD could potentially serve as an effective tool for delaying migraine chronification. In fact, characteristic MD foods, extra virgin olive oil and oily fish, rich in polyphenols and long-chain fatty acids, modulate inflammatory and oxidant pathways [28,37], which are deeply involved in the pathophysiology of migraine [38,39]. In addition, the MD is rich in vitamins C, E, B9, and carotenoids and therefore can counteract oxidative stress and lipid peroxidation, exerting cardioprotective and neuroprotective effects [5]. Finally, the MD seems capable of positively modulating the gut microbiota by increasing its α-diversity, the main indicator for describing the diversity and health of the gut microbiota, different from the saturated-fatty-acid-rich Western diet, which, on the contrary, favors gut dysbiosis and the production of gut-derived toxins [5,40,41,42].

Consistent with our findings, increased adherence to the MD has been associated with a lower frequency, duration, and severity of pain attacks [11,12].

Here, we did not find a relationship between adherence to the MD and migraine features in patients with low-frequency episodic migraine. However, the limited disability among the low-frequency episodic migraineurs might have prevented an eventual impact of adherence to the MD. Also, we did not find any relationship between diet patterns and other impact headache tools such as the MIDAS or VAS scores. However, we found that the HIT-6 scale was the best “impact index” in our population.

Along with adherence to the MD, we showed a significant relationship between migraine features and circadian sleep–wake rhythm disturbances. In particular, the presence of circadian sleep–wake disruption was significantly correlated with headache disability as assessed by the MIDAS score in patients with high-frequency episodic and chronic migraine. Consistent with the frequent comorbidity described between migraine and circadian sleep–wake disorders, approximately two-thirds (65%) of patients in this cohort suffered from some form of circadian disruption.

Sleep and migraine are bidirectionally linked due to shared neurobiological substrates [43], and while several studies support the evidence that people with migraine have a worse sleep quality, others indicate that sleep disorders might have a direct pathogenic role in migraine [44]. In particular, sleep deprivation might induce metabolic changes at the central level, reducing glycogen availability [45] and the clearance of various toxic proteins and waste metabolites through the glymphatic system [46], which could ultimately trigger neuroinflammation and could be consequently implied in migraine pathogenesis [46] and chronification [43]. Another possible link between migraine and sleep is pain medications. While several migraine drugs might affect sleep by either causing direct side effects or unmasking physiological alterations in sleep propensity seen as part of the headache syndrome, sleep deprivation might, in turn, diminish the effectiveness of headache treatments [43]. This mutual relationship has not been fully elucidated, and the broad range of different pharmacological therapies used in our sample, differentially modulating the sleep–wake cycle, did not allow us to homogenize this information.

Adiposity has a substantial impact on pain, possibly due to its neurometabolic effects [47]. However, it must be noted that the mean weight of our cohort was normal (BMI = 23.6 ± 3.5 kg/m^2^), possibly having nullified the potential relationships with susceptibility and severity to migraine.

Of relevance, we found that adherence to the MD and sleep quality were the only lifestyle characteristics analyzed in this study that were associated with headache severity and disability, as assessed by both migraine features and impact tools. Diet and sleep also appear to be connected by a complex relationship [12]. In fact, insufficient sleep can lead to overfeeding; metabolic impairment, with weight gain and obesity; and poor diet quality. On the contrary, lower adherence to the MD may worsen sleep quality and duration in otherwise healthy subjects [12]. Therefore, “guilt by association” may emerge between diet and sleep in our migraineur cohort, with a negative influence of lower adherence to the MD on the observed sleep dysfunction, although causation cannot be proven by such findings and should be verified by interventional studies. This implies that the potential health benefit in combatting the risk of chronic and severe migraine associated with adherence to the MD, as observed in our study, may depend on the grouping of healthy lifestyle features, which also include better sleep.

No relationships were found between pain characteristics and physical activity, as assessed by the IPAQ score, neither in the entire migraine cohort nor in the subgroups. However, although patients with migraine are reported to be less physically active compared to headache-free individuals, while low physical activity has been associated with a higher prevalence of migraine [48], we found that migraine sufferers were more physically active than the group of controls of the same age and sex. In fact, most of the patients in our migraine cohort were equally physically inactive or sufficiently active (both around 38%), while about one-quarter were very active. On the contrary, only 18% and 7% of the controls were considered sufficiently active and very active, respectively. The reasons underlying these discrepancies cannot be established here and may possibly refer to specific features of this migraine cohort, with patients choosing to engage in physical activity due to its putative benefits for the brain. From this point of view, the higher amount of physical activity found in the migraine cohort might be a consequence, rather than a cause, of migraine itself. However, the association between physical activity and migraine is far from fully elucidated. Indeed, although regular exercise may have a prophylactic effect on headache frequency, exercise can trigger migraine attacks [49]. Certainly, the relationship between migraine and the frequency, intensity, and type of exercise remains an open question that should be addressed in further dedicated studies, together with other specific features, such as water intake.

The study has some limitations due to its cross-sectional design, which did not allow for an evaluation of the longitudinal progression of the migraine characteristics over time, and the lack of a polysomnographic/actigraphic confirmation of sleep–wake disturbances. Additional limitations include its relatively small size, lack of socioeconomic status assessment, and insufficient evaluation of long-term MD adherence in the entire study population. Additionally, since the relationships between migraine, diet, sleep, and physical activity are complex and often bidirectional, another possible limitation of this study is reverse causation bias, where changes in dietary patterns, sleep, and amount of physical activity could be caused by migraine and not vice versa. Nevertheless, regardless of the fact that we found an association between migraine features, nutrition, and sleep, the design of this study prevents us from identifying potential causal relationships between these three conditions.

However, this study has several strengths, including the considerable sample size of migraine patients; the use of validated instruments, such as the HIT-6, MIDAS, PSQI, PREDIMED, and IPAQ scores; and the use of various possible confounders to adjust the regression models, including age, sex, and BMI, leading to unbiased risk estimates in this study.

## 5. Conclusions

In conclusion, we found that both the MD and sleep were tightly linked to the severity and disability of migraine, elucidating the role of lifestyle modification as a useful, practical, and highly accessible tool for delaying migraine chronification.

## Figures and Tables

**Table 1:** This table shows the main demographic and clinical characteristics of the study population.

	Migraine Patients (n = 170)	Controls (n = 100)	*p*-Value
Gender (M/F)	31/139	13/87	NS
Age (years)	44.4 ± 13.3	49.2 ± 15.7	NS
BMI (kg/m^2^)	23.6 ± 3.5	24.1 ± 4.2	NS
IPAQ (1/2/3)	65/65/40	38/18/7	*p* = 0.007 *
PREDIMED (1/2/3)	19/124/27	1/70/29	*p* < 0.001 *
Sleep–wake disturbances (Y/N)	110/60	63/37	NS
Migraine features			
Disease duration (years)	22.4 ± 15.3	/	/
MMDs	8.2 ± 7.6	/	/
Painkillers/month	5.3 ± 7.3	/	/
Mean duration of attacks (hours)	34.9 ± 20.7	/	/
VAS	8.1 ± 1.8	/	/
MIDAS	18.8 ± 22.5	/	/
HIT-6	57.6 ± 9.3	/	/
Pain localization (1/2/3/4)	50/19/18/82	/	/
Pain type (1/2/3)	79/3/88	/	/
UASs (Y/N)	19/151	/	/
Preventive treatments (n) (0/1/2/3/4)	41/87/31/7/4	/	/
Response to triptans (0/1/2/3)	6/26/75/63	/	/
>3 failed previous preventive treatments (Y/N)	12/158	/	/

MMDs, monthly migraine days; VAS, visual analogue scale; HIT-6 Headache Impact Test-6, MIDAS, Migraine Disability Assessment Scale, IPAQ, International Physical Activity Questionnaire: 1 = inactive, 2 = sufficiently active, 3 = very active; PREDIMED, PREvención con DIeta MEDiterránea: 1 = poor adherence, 2 = moderate adherence, 3 = good adherence; pain localization: 1 = fixed unilateral, 2 = alternating unilateral, 3 = unilateral/bilateral, 4 = bilateral; pain type: 1 = pulsating, 2 = sharp, 3 = other; UASs, unilateral autonomic symptoms; response to triptans: 0 = no, 1 = scarce, 2 = good, 3 = excellent). * Significant differences between groups.

**Table 2:** This table shows the main demographic and clinical characteristics of patients with low-frequency episodic migraine and patients with high-frequency episodic migraine plus chronic migraine.

	EM^LF^ Patients (n = 98)	EM^HF^ Patients + CM Patients (n = 72)	*p*-Value
Gender (M/F)	20/78	11/61	NS
Age (years)	43.1 ± 13.0	46.1 ± 13.6	NS
Disease duration (years)	23.3 ± 14.8	21.1 ± 15.1	NS
BMI (kg/m^2^)	23.7 ± 3.6	23.5 ± 3.4	NS
MMDs	3.3 ± 1.9	14.8 ± 12.9	*p* < 0.001 *
Symptomatics/month	2.4 ± 2.4	9.1 ± 9.6	*p* < 0.001 *
Mean attack duration	33.6 ± 21.1	36.6 ± 20.3	NS
VAS	7.7 ± 2.2	8.6 ± 1.1	*p* = 0.009 *
MIDAS	14.2 ± 19.1	25.0 ± 25.3	*p* = 0.001 *
HIT-6	53.56 ± 9.3	63.0 ± 5.8	*p* < 0.001 *
IPAQ (1/2/3)	39/35/24	26/30/16	NS
PREDIMED (1/2/3)	11/74/13	8/50/14	NS
Sleep–wake disturbances (Y/N)	62/36	48/24	NS
Pain localization (1/2/3/4)	36/9/9/43	14/10/9/39	NS
Pain type (1/2/3)	38/2/52	27/1/36	NS
UASs (Y/N)	11/87	8/64	NS
Preventive treatments (number) (0/1/2/3/4)	31/47/16/4/0	10/40/15/3/4	*p* = 0.019 *
Response to triptans (0/1/2/3)	2/8/43/45	4/18/32/18	*p* = 0.003 *
>3 failed previous preventive treatments (Y/N)	7/91	5/67	NS

MMDs, monthly migraine days; VAS, visual analogue scale; HIT-6 Headache Impact Test-6, MIDAS, Migraine Disability Assessment Scale, IPAQ, International Physical Activity Questionnaire: 1 = inactive, 2 = sufficiently active, 3 = very active; PREDIMED, PREvención con DIeta MEDiterránea: 1 = poor adherence, 2 = moderate adherence, 3 = good adherence; pain localization: 1 = unilateral, 2 = alternating unilateral, 3 = unilateral/bilateral, 4 = bilateral; pain type: 1 = pulsating, 2 = sharp, 3 = other; UASs, unilateral autonomic symptoms; response to triptans: 0 = no, 1 = scarce, 2 = good, 3 = excellent). * Significant differences between groups.

## Data Availability

The datasets generated during the analysis are available from the corresponding author upon reasonable request.
